# Species-Specific *COI* Primers for Rapid Molecular Identification of *Leucoptera malifoliella*

**DOI:** 10.3390/insects17080778

**Published:** 2026-07-28

**Authors:** Jiaqiang Zhao, Qiang Xu, Guijie Chi, Shengping Zhang, Ruitao Yu, Shihang Zhao, Qi Gao, Zhaohui Yang, Guoliang Xu

**Affiliations:** 1 Shijiazhuang Institute of Pomology, Hebei Academy of Agriculture and Forestry Sciences, Shijiazhuang 050061, China; jiaqiang_zhao@126.com (J.Z.); zspingyouxiang@haafs.org (S.Z.); wrightyu@haafs.org (R.Y.); zhaoshih3@haafs.org (S.Z.); qigao977@haafs.org (Q.G.); 2 Guangzhou Customs District Technology Center, Guangzhou 510623, China; xuqiangbj@foxmail.com; 3 Pingquan Municipal Bureau of Agriculture and Rural Affairs, Chengde 067500, China; chiguijie77@163.com; 4 Hebei Provincial Center of Agro-Product Safety and Quality, Shijiazhuang 050061, China

**Keywords:** *Leucoptera malifoliella*, molecular identification, DNA barcoding, rapid detection, SS-*COI* PCR

## Abstract

*Leucoptera malifoliella* (Lepidoptera: Lyonetiidae) is a quarantine pest of apple and other Rosaceae fruit trees whose range is expanding into new territories. Its minute size and morphological overlap with closely related Lyonetiidae make routine identification unreliable, especially for larvae and damaged specimens. We compared *COI* sequences from six common small Lepidoptera species found in orchards and designed the species-specific primer pair SXW-F/SXW-R. The resulting polymerase chain reaction (PCR) assay amplifies an ~500 base pairs (bp) fragment exclusively from *L. malifoliella*; no product was detected in any of five non-target species. The reaction tolerates annealing temperatures of 50–58 °C and consistently detects the target across all life stages (first- to third-instar larvae, pupae, adults) and all adult tissues tested (antennae, head-thorax, abdomen, wings, legs). Detection sensitivity reaches 0.03 ng/μL—approximately one-thousandth of the DNA content of a single adult. This is the first species-specific *COI* (SS-*COI*) method reported for *L. malifoliella*. It furnishes a rapid, specific, and sensitive diagnostic tool for quarantine inspection, field monitoring, and integrated pest management (IPM) programs.

## Simple Summary

*Leucoptera malifoliella* (Lepidoptera: Lyonetiidae) attacks apple and other Rosaceae fruit trees and is now subject to quarantine regulations in multiple countries. Adults are minute; larvae overlap morphologically with several congeners and other small orchard Lepidoptera, making routine identification slow and error-prone. We designed primers targeting the cytochrome coxidase subunit I (*COI*) gene and assembled a polymerase chain reaction (PCR) assay that amplifies an ~500 bp fragment exclusively from *L. malifoliella*. No cross-amplification occurred in any of five non-target species tested. The assay works across all developmental stages—from first-instar larvae through adults—and detects the target from a single leg or wing. Sensitivity reaches 0.03 ng/μL, roughly one-thousandth of the DNA in one adult. Results are available within 2–3 h. To our knowledge, this is the first species-specific *COI* (SS-*COI*) PCR method reported for *L. malifoliella*. Quarantine inspectors and growers can use it as a DNA-based alternative to morphology for screening orchard samples and for supporting integrated pest management (IPM) decisions.

## 1. Introduction

**Figure 1:**
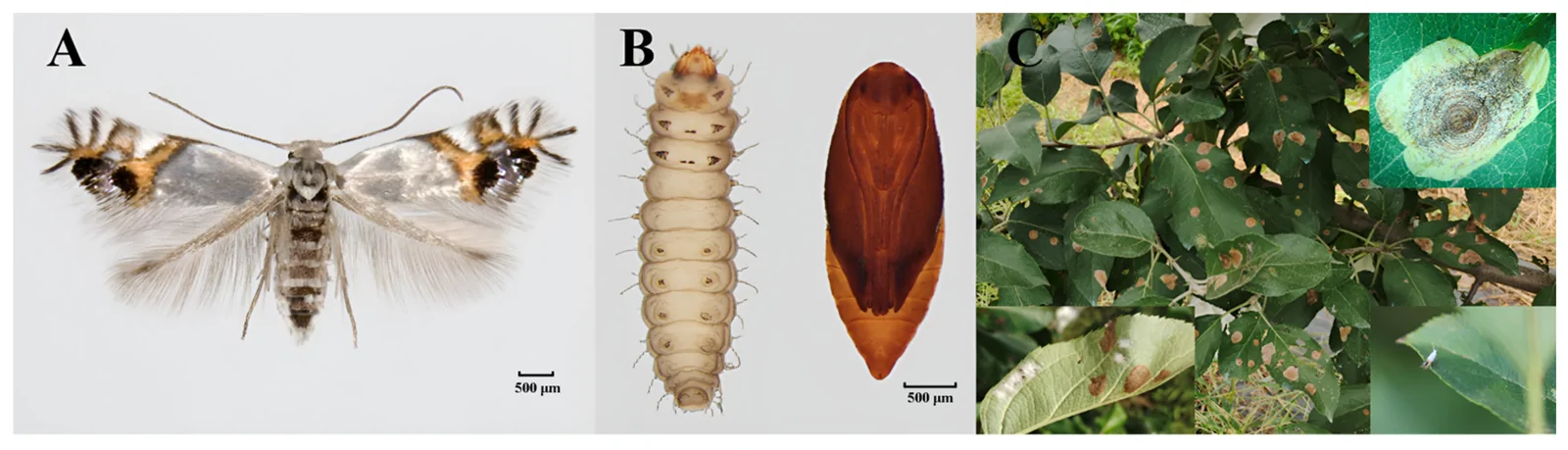
Morphology and damage symptoms of *Leucoptera malifoliella*. (**A**) Adult. (**B**) Larva and pupa. (**C**) Damage in an apple orchard, showing adults, silk cocoons, and blotch mines with concentric frass rings.

*Leucoptera malifoliella* (Costa, 1836) (Lepidoptera: Lyonetiidae)—the pear leaf blister moth or apple leaf miner—feeds on apple (*Malus domestica*) and other Rosaceae fruit trees across temperate Eurasia [1]. Its larvae are obligate leaf miners; they consume mesophyll tissue from within the leaf, producing circular blotch mines on the upper leaf surface with concentric frass rings (Figure 1). Heavy infestations—more than 10 mines per leaf—trigger premature leaf abscission, reduce photosynthetic capacity, and lower fruit quality, causing severe yield losses in commercial orchards [2,3]. This multivoltine species completes multiple overlapping generations annually under favorable conditions; larvae pass through three instars before pupating in silk cocoons on leaf undersides or in bark crevices. Generation overlap complicates population monitoring and precise control timing [4].

*Leucoptera malifoliella* is listed as an A1 quarantine pest by the European and Mediterranean Plant Protection Organization (EPPO) and classified as a high phytosanitary risk by the United States Department of Agriculture (USDA), reflecting concern over its potential introduction to North American apple-producing regions. Historically distributed across Europe, Central Asia, West Asia, and China [5], the species has expanded its range substantially in recent years. Soltani and Rahmouni [6] reported the first Tunisian record in 2024, documenting established populations at multiple sites in Kasserine Governorate in central-western Tunisia and identifying *L. malifoliella* as one of the most common apple orchard pests. In 2025, bimodal outbreak patterns were documented in the Shopian region of Kashmir, India [7], with population peaks in early spring (March–April) and late summer (August–September), followed by a decline in May–June. Among seven monitoring sites, Zainapora and Aglar showed the highest pest densities; only Sofipora exhibited a delayed peak. These invasion events highlight the growing phytosanitary threat from *L. malifoliella* and the urgent need for rapid identification tools to support early detection.

Traditional identification relies on morphological examination of adults and characteristic larval mines, but this approach has clear limits. *Leucoptera malifoliella* adults measure only 2–3 mm in body length with a wingspan of 7–9 mm; their features are readily confused with other leaf-mining moths such as *Phyllonorycter ringoniella*. Other small lepidopteran pests commonly found in apple orchards—including *Grapholita molesta*, *Adoxophyes orana*, *Cydia pomonella*, and *Endothenia oblongana*—share morphological similarities in larvae or pupae that non-specialists cannot reliably distinguish [8]. The process also demands experienced taxonomists and considerable time, and damaged, incomplete, or immature specimens (eggs, early-instar larvae, pupae) lack the diagnostic features needed for reliable identification [9,10]. These constraints limit the effectiveness of quarantine inspections, field monitoring, and integrated pest management (IPM) decision-making.

DNA barcoding offers a complementary approach to species identification. Proposed by Hebert et al. [11], it uses a standardized ~658 bp fragment of the mitochondrial cytochrome c oxidase subunit I (*COI*) gene as a universal species-level marker. The *COI* gene brings several practical advantages: maternal inheritance without recombination, absence of introns, a moderate evolutionary rate that generates enough variation for species discrimination while retaining conserved primer-binding regions, and universal primers that amplify the barcode region across multiple metazoan phyla [12]. Folmer et al. [13] designed the landmark primer pair LCO1490/HCO2198, which enabled broad-scale *COI* amplification across invertebrate phyla and remains foundational to barcoding protocols. Since then, the approach has been applied extensively in species identification, cryptic species discovery, biosecurity, and invasion biology. In Lepidoptera, Hajibabaei et al. [14] showed that *COI* barcodes reliably distinguish congeneric species of tropical butterflies and moths. Subsequent large-scale studies have validated *COI*-based identification across diverse lepidopteran lineages. Lopez-Vaamonde et al. [15] built a barcode library of 6791 *COI* sequences from 242 European Gracillariidae species and identified 93% of them using the Barcode Index Number (BIN) system. DNA barcoding has also traced invasion pathways in leaf-mining pests, as demonstrated for *Cameraria ohridella* and *Phyllonorycter issikii*. Beyond Gracillariidae, it has uncovered substantial undescribed diversity in microlepidopteran faunas of understudied regions such as Madagascar [16] and has served as a taxonomic verification framework in large-scale biodiversity projects [17]. The Barcode of Life Data Systems (BOLD) provides a centralized repository for barcode records, enabling sequence storage, species identification, and global data sharing [18].

Standard DNA barcoding nonetheless requires PCR amplification, Sanger sequencing, and bioinformatic analysis—procedures demanding specialized facilities, trained personnel, and several days [19]. These requirements restrict its use in resource-limited settings, field stations, and routine quarantine inspections where speed matters. Species-specific *COI* (SS-*COI*) PCR circumvents these constraints. The method uses primers targeting nucleotide positions conserved in the target species but divergent in all known close relatives, yielding a diagnostic DNA fragment unique to the target species [20]. Because SS-*COI* assays rely only on conventional PCR and agarose gel electrophoresis without sequencing, they cut per-sample costs and deliver results within 2–3 h [21]. They also work with degraded DNA from preserved specimens, eggs, and larval fragments, and require only basic molecular biology training for interpretation.

SS-*COI* markers have been developed for a range of agricultural and forest pests. Zhang et al. [22] designed primers for rapid identification of *Tuta absoluta*, an invasive pest of solanaceous crops. *Aleyrodes proletella*, a major pest of cruciferous vegetables, was targeted by species-specific *COI* markers established by Chen et al. [23]. For *Sirex noctilio*, an exotic invasive pest of pine forests, Sun et al. [24] reported SS-*COI* primers. Wang et al. [25] developed a multiplex SS-*COI* PCR method to distinguish *Phenacoccus manihoti* from its congener *P. herrerae*. More recently, Ji et al. [26] applied SS-*COI* markers to improve identification of *Lymantria dispar*—further evidence of the method’s reliability across insect taxa.

*L. malifoliella* can cause substantial orchard damage [27], and its recent range expansion into North Africa and South Asia has amplified phytosanitary concern. Yet no species-specific *COI* identification method exists for this pest—a gap that hampers IPM practitioners and phytosanitary agencies, especially in newly invaded regions where rapid identification is critical for containment. We address this gap by: (i) amplifying and sequencing the standard *COI* barcode region of *L. malifoliella* and selected orchard-associated lepidopteran species; (ii) identifying species-diagnostic nucleotide substitution sites through comparative sequence analysis; (iii) designing and validating species-specific forward and reverse primers for *L. malifoliella*; and (iv) optimizing a rapid SS-*COI* PCR protocol for quarantine and field monitoring. The resulting assay provides a practical, low-cost molecular tool for rapid *L. malifoliella* identification, complementing morphological methods and supporting IPM and phytosanitary decision-making.

## 2. Materials and Methods

### 2.1. Insect Samples

One hundred ninety specimens representing six small-bodied lepidopteran species were collected from apple orchards in Hebei Province, China, between May 2024 and September 2025. The sampled species included *Leucoptera malifoliella*, *Phyllonorycter ringoniella* (Matsumura, 1931) (Lepidoptera: Gracillariidae), *Grapholita molesta* (Busck, 1916) (Lepidoptera: Tortricidae), *Adoxophyes orana* (Fischer von Röslerstamm, 1834) (Lepidoptera: Tortricidae), *Cydia pomonella* (Linnaeus, 1758) (Lepidoptera: Tortricidae), and *Endothenia oblongana* (Haworth, 1811) (Lepidoptera: Tortricidae). Collection details (sampling locations, dates, and geographic coordinates) are summarized in Table 1, and the adult morphological photographs are shown in Figures S1 and S2.

All specimens were identified to species based on established morphological characteristics, including wing patterns, body length, and genitalia features where necessary [28]. Species identity was then confirmed by DNA barcoding of the mitochondrial cytochrome c oxidase subunit I (*COI*) gene and nucleotide–nucleotide BLAST queries against the NCBI GenBank database (https://blast.ncbi.nlm.nih.gov/Blast.cgi, accessed on 1 July 2026). Specimens were preserved in absolute ethanol (≥99.7%) and stored at −20 °C until DNA extraction.

### 2.2. DNA Extraction

We extracted total genomic DNA from specimens using the OMEGA Tissue DNA Kit (OMEGA Bio-Tek, Norcross, GA, USA) according to the manufacturer’s instructions with slight modifications [29]. Because of the minute body size of *L. malifoliella*, each extraction used either a single adult or 3–5 larvae. Insect tissues were ground to a fine powder in liquid nitrogen with a sterile micro-pestle, then transferred to a 1.5 mL microcentrifuge tube containing 180 μL of Buffer ATL and 20 μL of proteinase K (20 mg/mL). The mixture was incubated at 56 °C with intermittent agitation until complete digestion. Buffer AL (200 μL) was added, and incubation continued at 56 °C for 10 min. Absolute ethanol (200 μL, 96–100%) was added to the lysate, and the entire mixture was transferred to a HiBind DNA column. The column was washed sequentially with 500 μL of Buffer AW1 and 500 μL of Buffer AW2. Purified DNA was eluted twice with 200 μL of Buffer AE (10 mM Tris-Cl, 0.5 mM EDTA, pH 9.0) to maximize recovery. Concentration and purity were assessed on a NanoDrop One spectrophotometer (Thermo Fisher Scientific, Waltham, MA, USA). We retained samples with an A260/A280 ratio of 1.8–2.0 and an A260/A230 ratio > 2.0 for downstream analysis. All DNA extracts were stored at −20 °C.

### 2.3. Universal COI Sequencing and Phylogenetic Analysis

A ~658 bp fragment of the mitochondrial *COI* gene was amplified using the universal forward primer LCO1490 (5′-GGTCAACAAATCATAAAGATATTGG-3′) and reverse primer HCO2198 (5′-TAAACTTCAGGGTGACCAAAAAATCA-3′) [13]. PCR was performed in 25 μL reactions containing 12.5 μL of 2× Taq PCR Master Mix (TIANGEN Biotech, Beijing, China), 1 μL of each primer (10 μmol/L), 1 μL of template DNA (~30–50 ng), and nuclease-free double-distilled water (ddH_2_O) to volume. The thermal cycling program comprised an initial denaturation at 94 °C for 5 min; 35 cycles of 94 °C for 30 s, 48 °C for 30 s, and 72 °C for 1 min; and a final extension at 72 °C for 10 min. Each PCR batch included a negative control (ddH_2_O substituted for template DNA).

PCR products (5 μL) were separated on a 1.5% agarose gel in 1× Tris-acetate-EDTA (TAE) buffer (40 mM Tris-acetate, 1 mM EDTA, pH 8.0), with DL2000 DNA Marker (Takara Bio, Kusatsu, Japan) as the reference. Gels were stained with GelRed nucleic acid gel stain (Biotium, Fremont, CA, USA) and visualized under UV transillumination. Successfully amplified products were purified using the TIANGEN Gel Extraction Kit (TIANGEN Biotech) and submitted for bidirectional Sanger sequencing to Beijing Tsingke Biotech Co., Ltd. (Beijing, China) on an ABI 3730xl DNA Analyzer (Applied Biosystems, Foster City, CA, USA).

Raw sequencing chromatograms were assembled and edited in BioEdit v7.2.5. We generated consensus sequences from forward and reverse reads and queried them against the NCBI GenBank non-redundant nucleotide database via BLASTn to confirm species identity. Phylogenetic analysis was conducted in MEGA 12 [30]. Sequence alignment employed the ClustalW algorithm with default parameters. A neighbor-joining (NJ) tree was constructed under the Kimura 2-parameter (K2P) model; node support was evaluated by 1000 bootstrap replicates, with values ≥ 50% indicated at branch nodes. Pairwise genetic distances among the six species were calculated using both *p*-distance and K2P models and visualized as a heatmap.

### 2.4. Primer Design

We compared *COI* sequences of all six lepidopteran species, aligning them with both the ClustalW and MUSCLE algorithms in MEGA 12 to identify regions conserved in *L. malifoliella* but variable in the other five species. Candidate primers were designed in Primer Premier 5.0 with the following parameters: length 18–25 nt, GC content 40–60%, melting temperature (Tm) 50–60 °C, and expected amplicon size 200–500 bp [31]. Self-complementarity, hairpin potential, and 3′-end stability were evaluated to ensure robust amplification.

**Figure 2:**
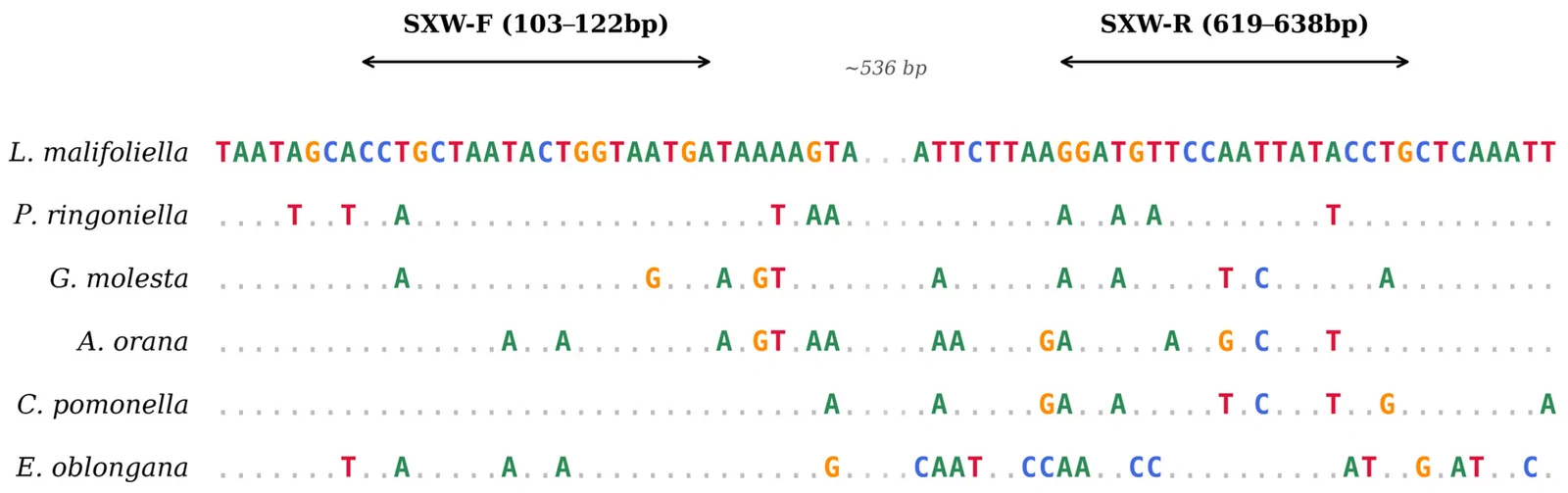
Sequence alignment of mitochondrial *COI* gene fragments from six lepidopteran species and design of species-specific primers for *L. malifoliella*. Variable nucleotide sites of non-target species are marked in different colors: red = T, green = A, blue = C, orange = G. Arrows indicate the positions of the species-specific primers SXW-F and SXW-R.

This analysis yielded a species-specific primer pair targeting a ~500 bp region of the *L. malifoliella COI* gene (Figure 2): SXW-F (5′-CCTGCTAATACTGGTAATGA-3′) and SXW-R (5′-CAGGTATAATTGGAACATCC-3′). Primer binding sites were validated in silico to confirm species-specific mismatches at the 3′ end of non-target species. To extend this in silico validation to congeners not represented in our specimen panel, both primer-binding sites were additionally aligned against all publicly available COI records of the family Lyonetiidae, and a database-wide screen was performed to confirm that no sequence other than *L. malifoliella* possesses both binding sites in the correct orientation and spacing.

### 2.5. Specificity Testing

Genomic DNA from all six lepidopteran species served as template to test whether the SXW-F/SXW-R primer pair specifically identified *L. malifoliella*. DNA from *L. malifoliella* served as the positive control; ddH_2_O replaced template in the negative control. PCR conditions were identical to those for universal *COI* amplification (Section 2.3), except that the universal primers were replaced with SXW-F and SXW-R. Products were analyzed by 1.5% agarose gel electrophoresis as described in Section 2.3. Positive amplicons were purified, submitted for bidirectional Sanger sequencing, and their identity confirmed by BLASTn. Unless otherwise specified, all PCR assays described in Section 2.5, Section 2.6, Section 2.7, Section 2.8, Section 2.9 and Section 2.10 were performed in three independent replicates on different days, each including positive (*L. malifoliella* DNA) and negative (ddH_2_O) controls.

### 2.6. Annealing Temperature Optimization

Gradient PCR was performed with *L. malifoliella* genomic DNA to determine the optimal annealing temperature (Ta) for SXW-F/SXW-R. Eight temperatures were tested: 50, 52, 54, 55, 56, 58, 59, and 60 °C. Each 25 μL reaction was prepared as described in Section 2.3. Products were separated on a 1.5% agarose gel, and band intensity, clarity, and specificity were assessed visually. The temperature producing the brightest, clearest single band without primer dimers or non-specific products was adopted for all subsequent assays.

### 2.7. Developmental Stage Testing

Genomic DNA was extracted independently from first- to third-instar larvae, pupae, and adults of *L. malifoliella* to evaluate the species-specific *COI* (SS-*COI*) PCR assay across developmental stages. PCR amplification with SXW-F/SXW-R followed the optimal conditions from Section 2.6; success was assessed by agarose gel electrophoresis.

### 2.8. Adult Tissue DNA Testing

The robustness of the SS-*COI* assay was tested using DNA from discrete body tissues of *L. malifoliella* adults: antennae, head and thorax (combined), abdomen, wings, and legs (Figure S3). Each tissue type was dissected from individual adults, extracted separately using the modified protocol in Section 2.2, and amplified under the optimized SXW-F/SXW-R.

### 2.9. Sensitivity Testing

The detection limit of the SS-*COI* PCR assay was determined using 10-fold serial dilutions of *L. malifoliella* genomic DNA. Starting DNA concentration was 30 ng/μL. Dilutions were prepared in nuclease-free ddH_2_O as follows: 10× (3 ng/μL), 100× (0.3 ng/μL), 1000× (0.03 ng/μL), 10,000× (3 pg/μL), and 100,000× (0.3 pg/μL). Each dilution was amplified with SXW-F/SXW-R under optimized conditions. The detection limit was the lowest concentration yielding a clear, specific band on agarose gel.

### 2.10. In Silico Primer-Site Conservation Analysis and Geographic Population Validation

To assess primer-site conservation across the currently documented global range, all publicly available *L. malifoliella COI/COX1* records were retrieved from NCBI GenBank and BOLD on 15 July 2026. NCBI returned 52 accession-level records; BOLD returned 78 species-level records, from which 68 *COI* records were retained after excluding non-*COI* marker fragments. The combined audit contained 120 source-specific records and 68 unique records after cross-database deduplication. These records represent China, India, Italy, Finland, France, Russia (Altai), and additional European countries, and include 43 deduplicated Indian records, of which 37 were collected in the Kashmir Valley in 2023 (Srinagar, Shopian, Wachi/Shopian–Wachi, Anantnag, Pulwama, and Kulgam). The complete mitogenomes OR438296 were used as Chinese references.

Two additional geographic populations of *L. malifoliella* from China (Chengde (118.60595, 40.87313), Hebei Province; Tai’an (117.114268, 36.19869), Shandong Province) were tested with the SXW-F/SXW-R primer pair under the optimized conditions described in Section 2.6. In parallel, a synthetic double-stranded template corresponding to the T-haplotype amplicon—designed from the Italian record PX980002, in which the terminal 3′ base of the SXW-R binding site is T rather than C—was amplified under the same conditions. Products were analyzed by 1.5% agarose gel electrophoresis as described in Section 2.3.

## 3. Results

### 3.1. COI Sequence Analysis and Phylogenetic Relationships

**Figure 3:**
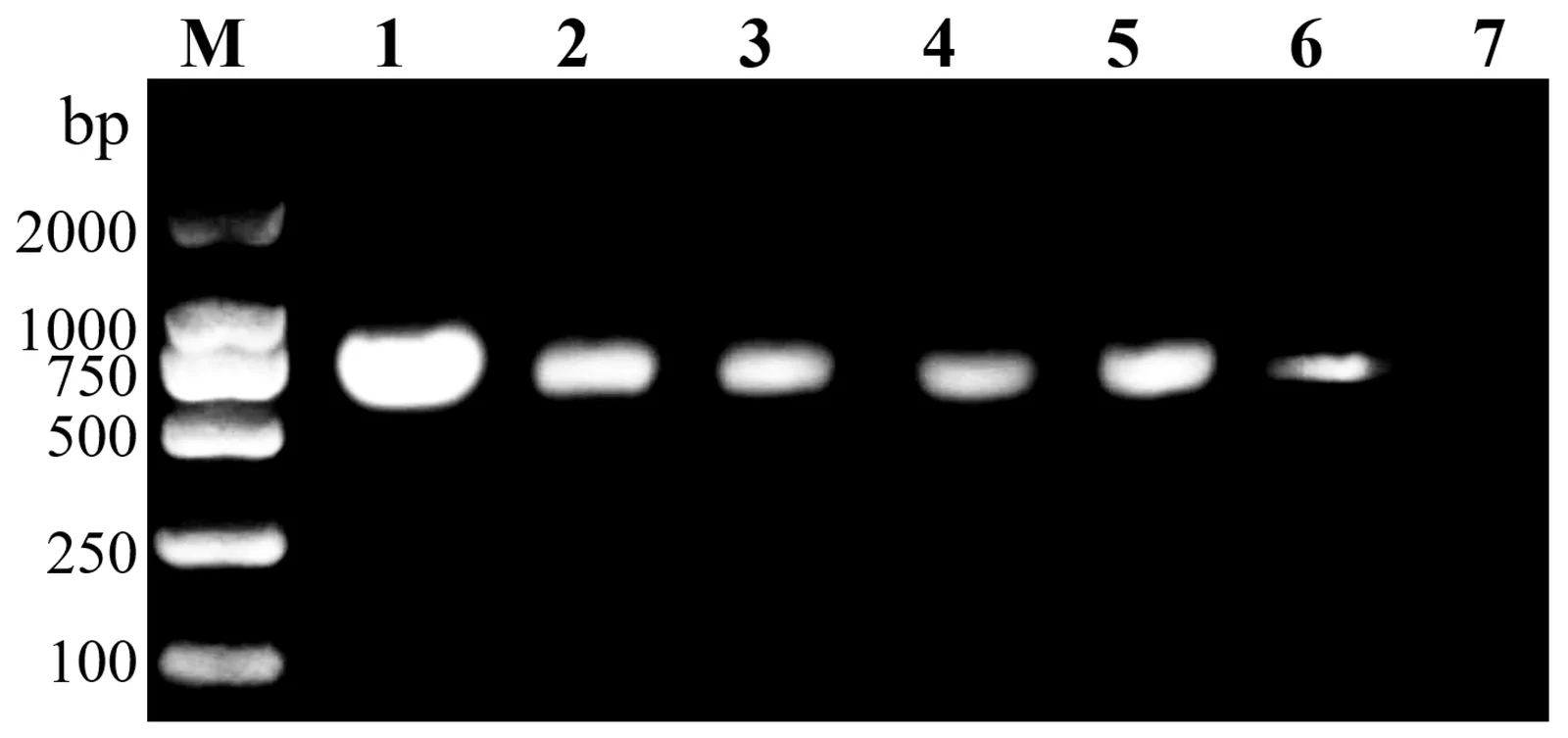
Agarose gel electrophoresis of *COI* gene fragments amplified from six lepidopteran species using the universal primers LCO1490 and HCO2198. M, DNA marker (DL2000); Lane 1, *Leucoptera malifoliella*; Lane 2, *Phyllonorycter ringoniella*; Lane 3, *Grapholita molesta*; Lane 4, *Cydia pomonella*; Lane 5, *Adoxophyes orana*; Lane 6, *Endothenia oblongana*; Lane 7, negative control.

Universal primers LCO1490 and HCO2198 amplified a ~658 bp fragment of the cytochrome c oxidase subunit I (*COI*) gene from all six lepidopteran species (Figure 3). BLASTn analysis confirmed the identity of all sequences, with 100% query coverage and ≥99% similarity to corresponding reference sequences in the database. The generated sequences were deposited in the National Center for Biotechnology Information (NCBI) GenBank database.

An NJ tree based on K2P distances (1000 bootstraps) resolved the six species into six distinct, well-supported clades (Figure S4). *L. malifoliella* formed an independent monophyletic clade (93% bootstrap), clearly separated from all non-target species; *P. ringoniella* also formed its own clade, while the four tortricid species clustered together (56%), with *G. molesta* and *C. pomonella* as sister species and *E. oblongana* basal. Pairwise genetic distances corroborated these patterns (Figure S4B): interspecific *p*-distance and K2P distance ranged from 0.0931/0.0994 (*G. molesta*–*C. pomonella*) to 0.1637/0.1847 (*L. malifoliella*–*A. orana*), all well above the 2% species-delimitation threshold. Distances between *L. malifoliella* and the five non-target species were 0.1366–0.1637 (*p*-distance; mean 0.1508) and 0.1508–0.1847 (K2P; mean 0.1684). This high interspecific divergence provides abundant species-specific nucleotide substitution sites suitable for diagnostic primer design.

Primer-binding sites were conserved across all globally available *L. malifoliella COI* records. The SXW-F binding site was 100% invariant across every record examined. The SXW-R binding site was likewise invariant except for the terminal 3′ base, which is C in the complete-mitogenome references (GenBank accession OR438296) and in our validated Chinese populations, and T in all European and Indian field-barcode records; no other primer-site polymorphism was detected. Thus, across all sequence data currently available worldwide, the primer sites of the assay are essentially invariant at the species level, and Indian haplotypes are indistinguishable from European ones at both sites.

### 3.2. Primer Specificity Validation

**Figure 4:**
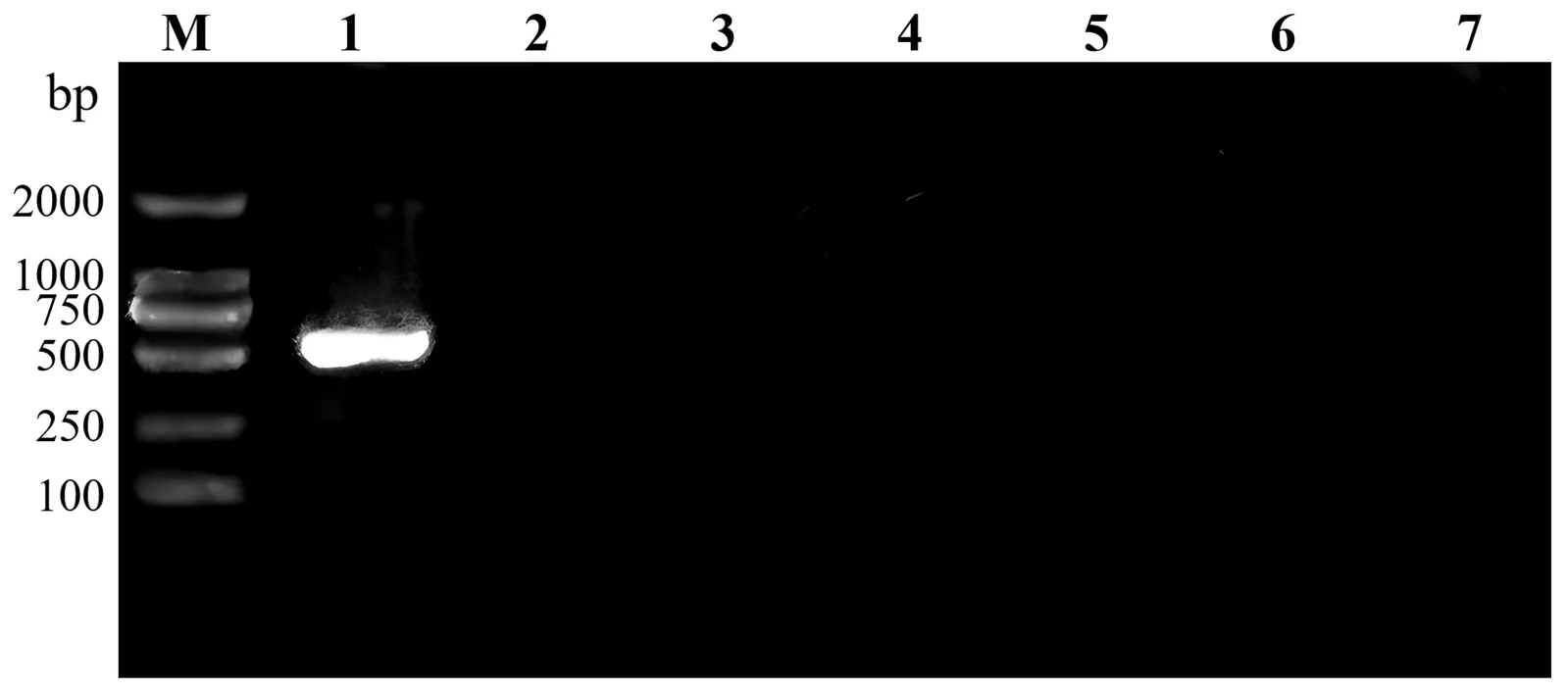
Specificity evaluation of the species-specific primer pair SXW-F/SXW-R against six lepidopteran species. A clear ~500 bp amplification band is present exclusively in *L. malifoliella* (Lane 1). No amplification was observed in any non-target species. M, DNA marker (DL2000); Lane 1, *Leucoptera malifoliella*; Lane 2, *Phyllonorycter ringoniella*; Lane 3, *Grapholita molesta*; Lane 4, *Cydia pomonella*; Lane 5, *Adoxophyes orana*; Lane 6, *Endothenia oblongana*; Lane 7, negative control.

PCR with the SXW-F/SXW-R primer pair on DNA templates from the six lepidopteran species specifically amplified a ~500 bp fragment exclusively from the *L. malifoliella* template DNA (Figure 4). No amplification product was detected in the remaining five non-target species even after 35 PCR cycles. The negative control with double-distilled water (ddH_2_O) as template likewise produced no amplification product, confirming the absence of contamination. Bidirectional sequencing of the *L. malifoliella* positive amplicon confirmed 100% identity with the corresponding *COI* sequence of *L. malifoliella* deposited in GenBank (accession number PX456999). The SXW-F/SXW-R primer pair showed absolute specificity for *L. malifoliella* and did not cross-react with any of the tested non-target species, including co-occurring orchard tortricids.

### 3.3. Effect of Annealing Temperature on PCR Amplification

**Figure 5:**
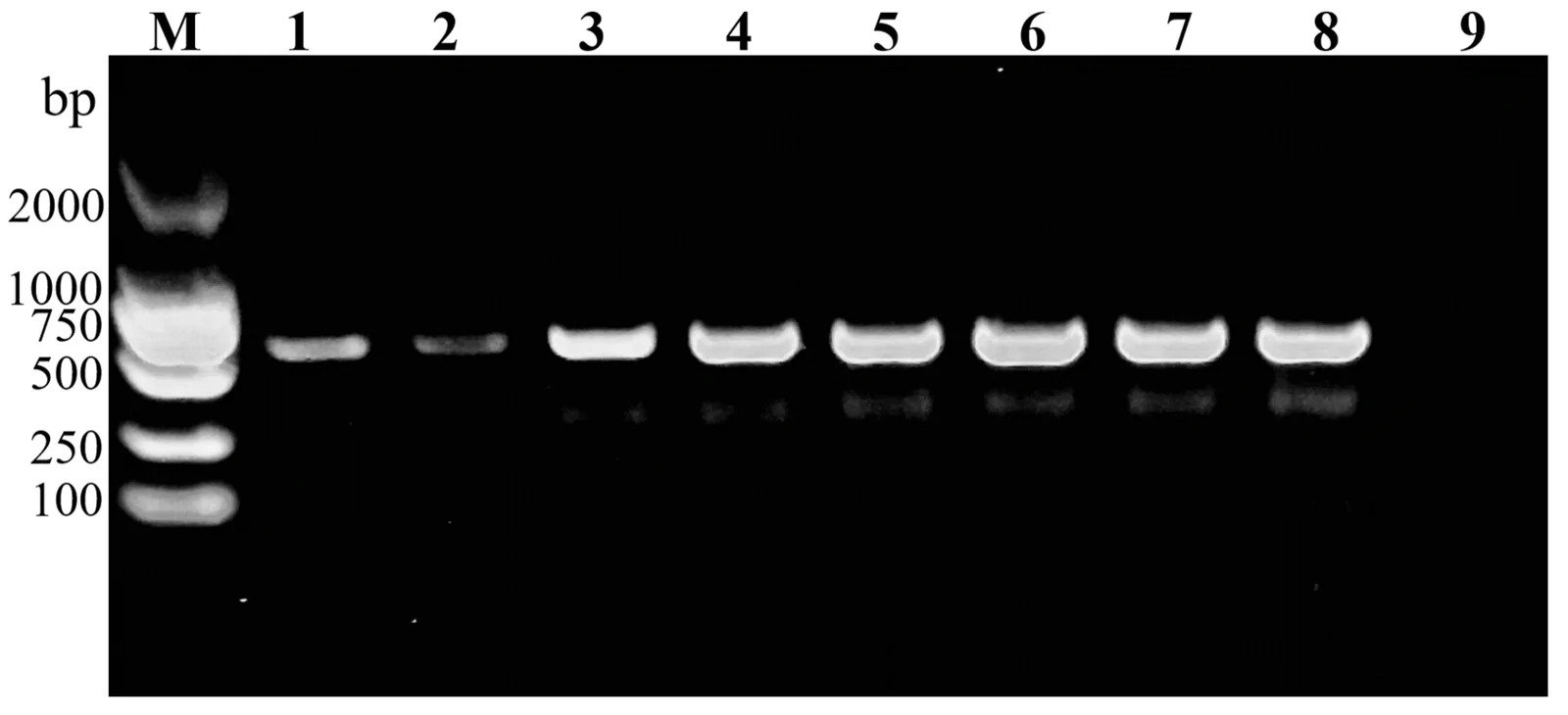
Gradient PCR analysis of the SXW-F/SXW-R primer pair across annealing temperatures ranging from 50 °C to 60 °C. M, DNA marker (DL2000); Lanes 1–8 correspond to annealing temperatures of 60 °C, 59 °C, 58 °C, 56 °C, 55 °C, 54 °C, 52 °C, and 50 °C; Lane 9, negative control.

Gradient PCR across eight annealing temperatures was performed to determine the optimal annealing temperature for the SXW-F/SXW-R primer pair. Successful amplification of the expected ~500 bp fragment was observed at all tested temperatures from 50 °C to 60 °C (Figure 5). Clear, bright amplification bands were obtained in the temperature range of 50 °C to 58 °C, indicating robust and efficient primer-template binding within this range. At annealing temperatures of 59 °C to 60 °C, amplification bands remained clearly visible but with markedly reduced intensity, indicating a gradual decline in annealing efficiency as the temperature increased. No non-specific amplification products or primer dimer bands were observed at any of the tested temperatures. Based on these results, the optimal annealing temperature range for the SXW-F/SXW-R primers was determined to be 50–58 °C, with 56 °C recommended as the standard annealing temperature for routine diagnostic applications.

### 3.4. Developmental Stage Applicability

**Figure 6:**
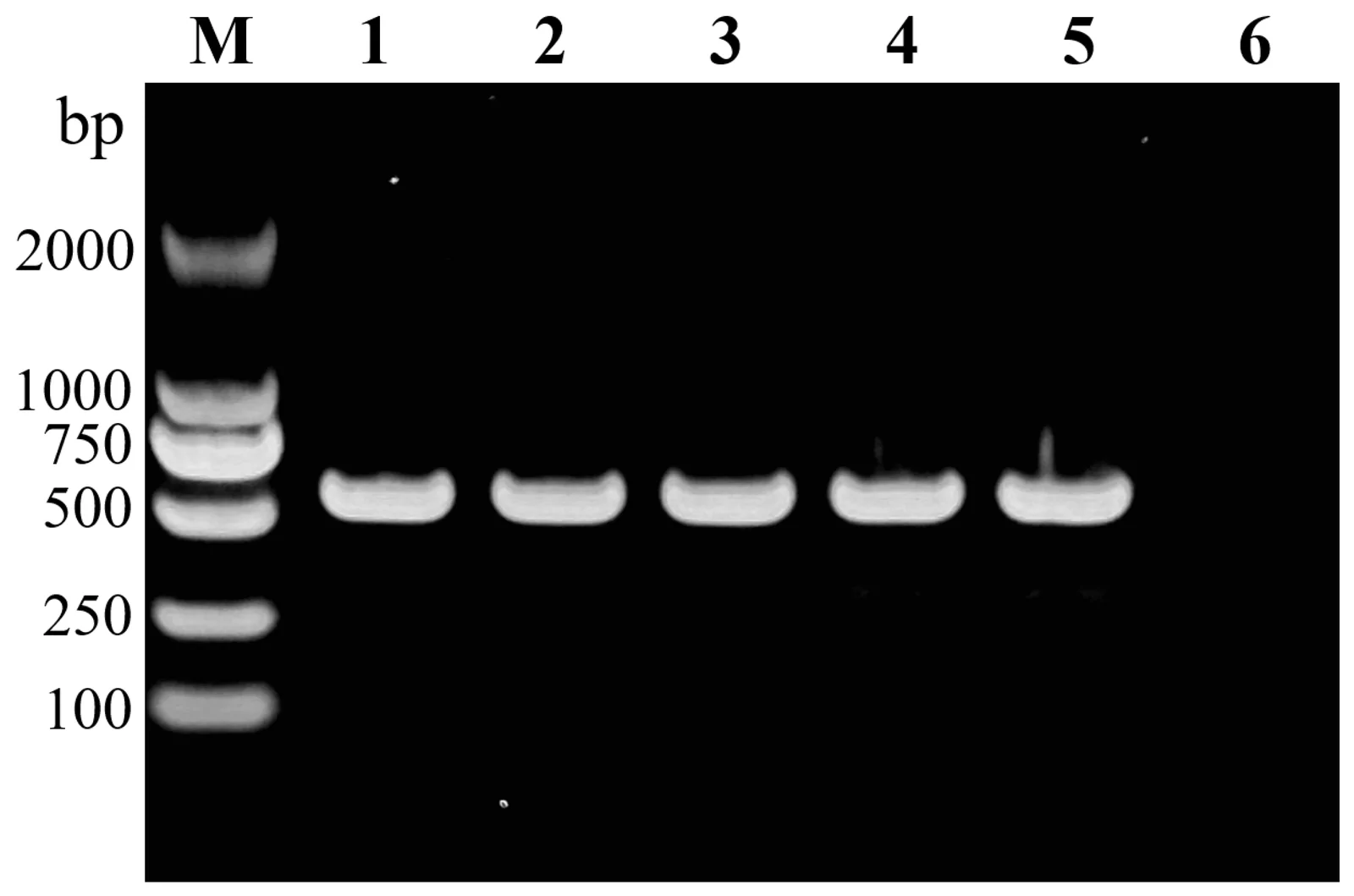
Amplification of the *L. malifoliella*-specific *COI* fragment from five different developmental stages using the SXW-F/SXW-R primer pair. M, DNA marker (DL2000); Lane 1, first-instar larva; Lane 2, second-instar larva; Lane 3, third-instar larva; Lane 4, pupa; Lane 5, adult; Lane 6, negative control.

The SXW-F/SXW-R primer pair was tested on DNA templates from five distinct developmental stages of *L. malifoliella*. Clear ~500 bp amplification bands were successfully obtained from all five developmental stages (Figure 6), with similar band intensity across stages. The species-specific PCR assay is not affected by the developmental stage of the specimen for identification of *L. malifoliella*, including early-instar larvae most frequently encountered in field surveys and monitoring programs. The ability to amplify target DNA from tiny first-instar larvae is advantageous for early detection and timely implementation of management strategies.

### 3.5. Adult Tissue Applicability

**Figure 7:**
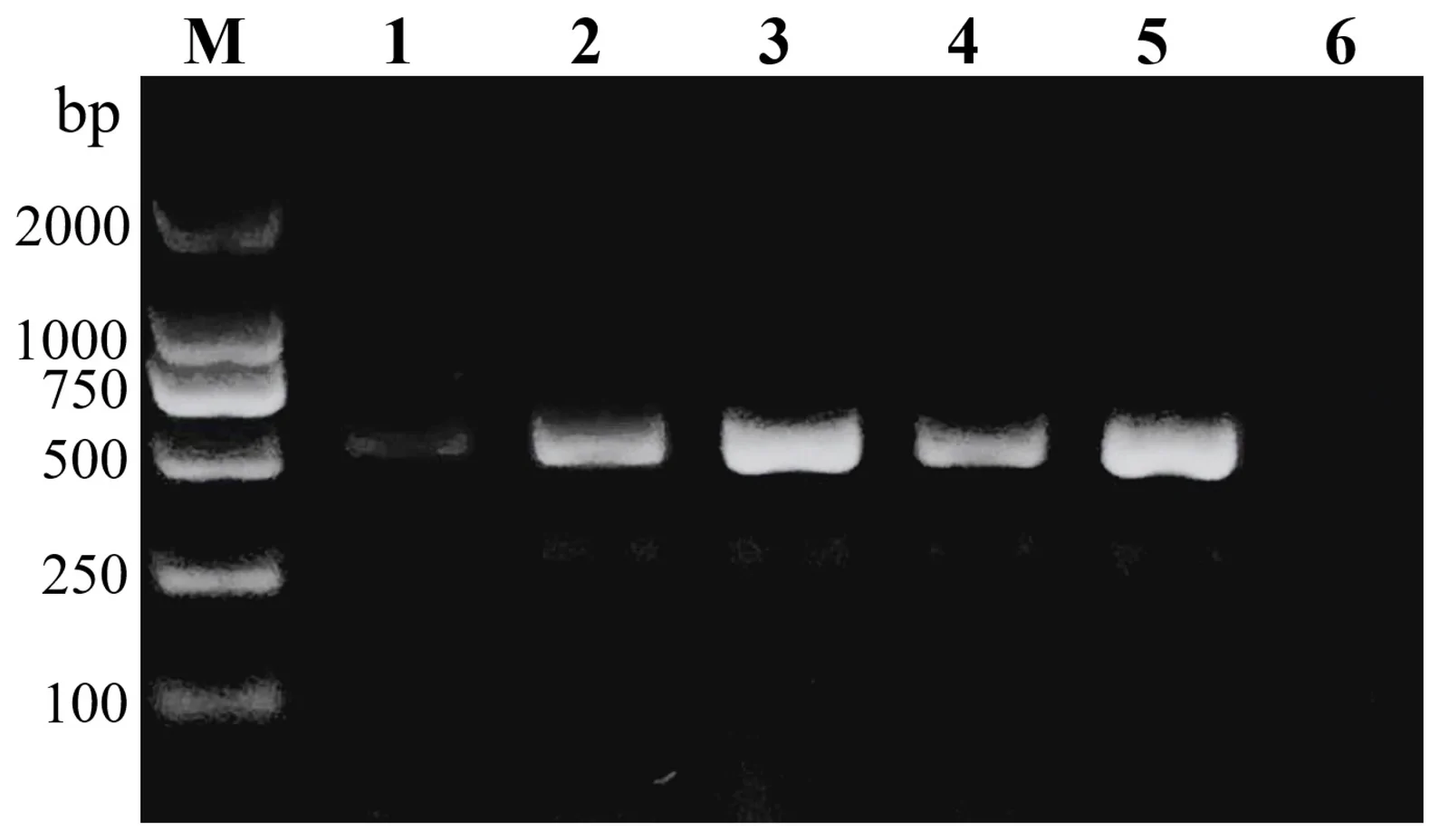
Tissue applicability testing of the SXW-F/SXW-R primer pair using DNA extracted from five different adult tissue types of *L. malifoliella*. M, DNA marker (DL2000); Lane 1, antenna; Lane 2, head–thorax; Lane 3, abdomen; Lane 4, wing; Lane 5, leg; Lane 6, negative control.

To test whether the assay works with minimal starting material, PCR amplification was performed using DNA extracted from five different adult tissue types: antennae, head-thorax, abdomen, wings, and legs. All five tissue types produced clear ~500 bp amplification bands (Figure 7), confirming that the species-specific primers are compatible with multiple tissue sources. Band intensity varied slightly among tissue types: abdomen and head-thorax samples yielded the strongest signals, consistent with higher mitochondrial DNA content in metabolically active tissues, whereas antennae and wing tissues produced relatively weaker but still clearly visible bands. The diagnostic assay is applicable to damaged or incomplete specimens, requiring only trace amounts of tissue for reliable species identification.

### 3.6. Detection Sensitivity

**Figure 8:**
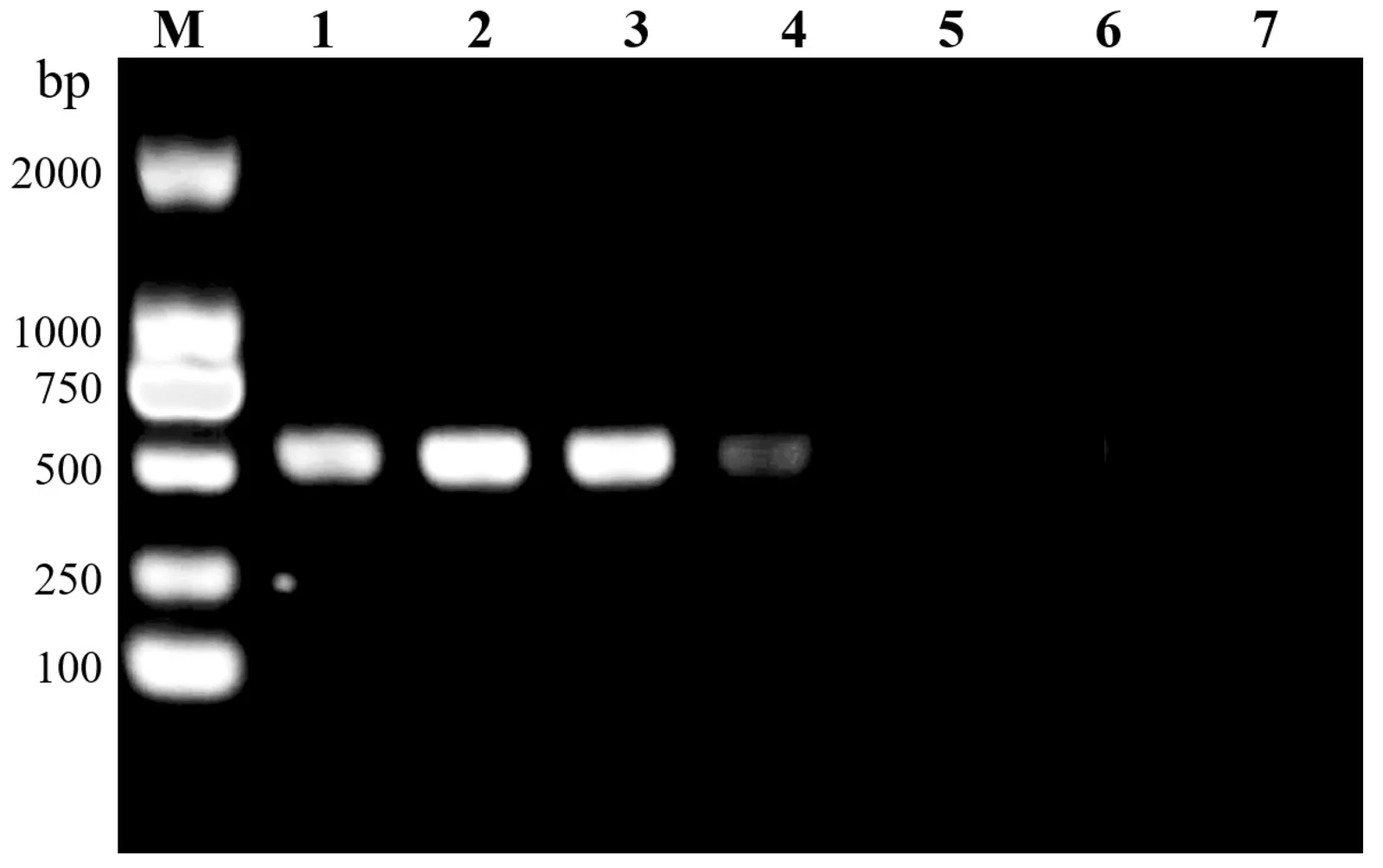
Sensitivity analysis of the SXW-F/SXW-R primer pair using 10-fold serial dilutions of *L. malifoliella* genomic DNA. M, DNA marker (DL2000); Lane 1, undiluted (30 ng/μL); Lane 2, 10-fold dilution (3 ng/μL); Lane 3, 100-fold dilution (0.3 ng/μL); Lane 4, 1000-fold dilution (0.03 ng/μL, 30 pg/μL); Lane 5, 10,000-fold dilution (3 pg/μL); Lane 6, 100,000-fold dilution (0.3 pg/μL); Lane 7, negative control.

Ten-fold serial dilutions of *L. malifoliella* genomic DNA were used to determine the detection sensitivity of the SXW-F/SXW-R primer pair. The initial DNA stock concentration (extracted from a single adult specimen) was 30 ng/μL. Serial dilutions were prepared and subjected to PCR amplification under standard conditions. Strong amplification was observed in the undiluted (30 ng/μL) and 10-fold diluted (3 ng/μL) samples (Figure 8). Clear amplification bands were also obtained at 100-fold dilution (0.3 ng/μL) and 1000-fold dilution (0.03 ng/μL, equivalent to 30 pg/μL). No amplification product was detected at 10,000-fold dilution (3 pg/μL) or 100,000-fold dilution (0.3 pg/μL). The minimum detection limit of the SXW-F/SXW-R primer pair was 0.03 ng/μL (30 pg/μL), corresponding to approximately one-thousandth of the total DNA extractable from a single adult *L. malifoliella*.

### 3.7. Validation on Geographically Distinct Populations and the T-Haplotype Template

**Figure 9:**
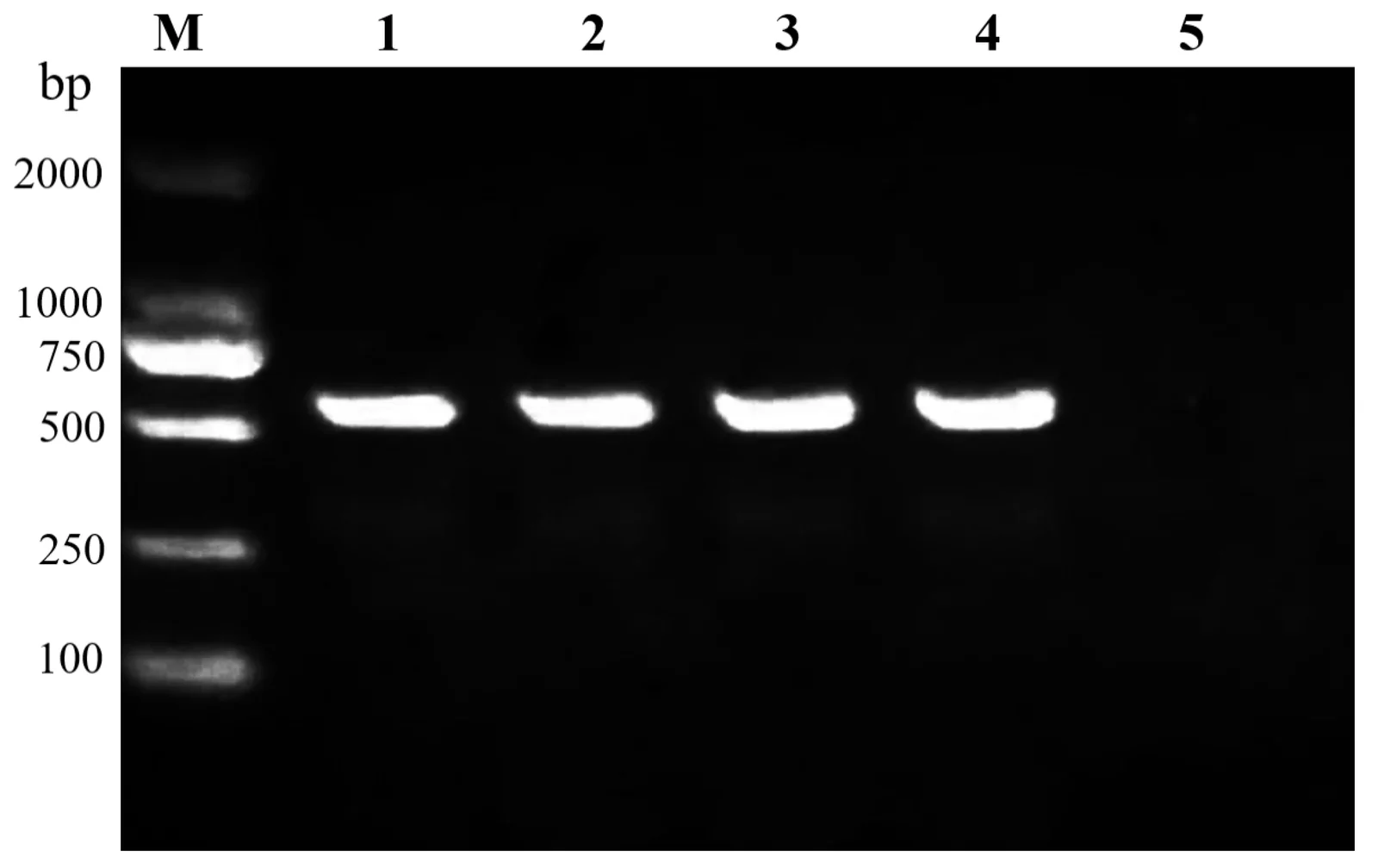
Validation of primer performance on geographically distinct *Leucoptera malifoliella* populations and the T-haplotype amplicon template. M, DNA marker (DL2000); Lanes 1 and 2, Chengde and Tai’an population templates amplified with the SXW-F/SXW-R primer pair; Lanes 3 and 4, synthetic T-haplotype template amplified with the same primers; Lane 5, negative control.

The SXW-F/SXW-R primer pair amplified the expected ~500 bp fragment cleanly from both additional geographic populations (Chengde and Tai’an; Figure 9). The synthetic double-stranded template corresponding to the T-haplotype amplicon likewise produced the specific ~500 bp band, demonstrating that the single 3′-terminal C/T dimorphism in the SXW-R binding site does not prevent amplification. Identical target bands were therefore obtained from all population samples and from the T-haplotype template, confirming that the nucleotide variation defining the T-haplotype does not alter the electrophoretic banding pattern.

## 4. Discussion

Accurate species identification underpins every control decision and quarantine ruling in orchard pest management and cross-border trade [32]. *Leucoptera malifoliella* adults are tiny (5.7–7.7 mm wingspan). Larvae mine inside leaves, hidden from view. Several congeneric species share overlapping morphological characters. Together these traits make visual identification error-prone and slow, which in turn delays control and trips up border inspections. A rapid, deployable molecular assay usable at the grassroots level would fill an immediate practical gap for monitoring this pest [33,34].

We designed species-specific *COI* (SS-*COI*) primers from mitochondrial *COI* sequence data and built a conventional PCR assay that runs from DNA extraction to gel interpretation in 2–3 h. The primers showed clean specificity across all geographic populations and life stages, a detection floor of 30 pg, and 100% accuracy on field-collected specimens, with no cross-amplification against the non-target species tested.

### 4.1. Practical Needs and Technical Bottlenecks for Rapid and Precise Identification of L. malifoliella

*L. malifoliella* ranks among the most destructive leaf-mining pests in Eurasian temperate apple orchards. Its larvae are obligate miners; circular concentric mines can occupy 3.4–4.6% of individual leaf area, and >40 mines per leaf will trigger premature defoliation [6]. The pest’s range is still expanding—confirmed establishment in India (2023) and Tunisia (2024), probably hitchhiking on nursery stock or fresh fruit—ratcheting up the pressure on apple industries worldwide [6]. Port quarantine services and field-response teams now face tighter deadlines for accurate identification.

Yet accurate ID of *L. malifoliella* has long been bottlenecked by biology and logistics. Adults are minute, forewings are silvery-white with a metallic sheen, and diagnostic characters wear easily [35,36]. Early-instar larvae lie deep in leaf mesophyll; pupae remain inside mines. Both stages overlap morphologically with congeners and with other Lyonetiidae leaf miners [37,38]. Traditional diagnosis requires genitalia dissection and comparison with voucher specimens, which demands intact material, trained taxonomists, and several days—time that ports and field stations do not have. Image recognition has emerged as an alternative [39,40], but small-target detection remains imprecise, damaged specimens and cluttered backgrounds confound algorithms, and the technology still cannot stand alone as a definitive diagnostic tool.

DNA barcoding with the mitochondrial *COI* gene offers a more reliable route [41]. *COI* is maternally inherited, conserved within species, and variable between them. Hebert et al. [42] formalized the species-delimitation framework using interspecific genetic distance. We compared *COI* sequences of *L. malifoliella* with those of closely related species and found K2P distances of 0.1508–0.1847—well above the interspecific discrimination threshold for Lepidoptera. That divergence gave us the molecular footing for primer design. The resulting SS-*COI* assay needs no bidirectional sequencing or database lookup. It works on eggs, larvae of any instar, pupae, adults, and even minute tissue fragments such as a single leg or antenna—circumventing the chronic problem of identifying damaged or early-stage material. It outperforms standard barcoding on speed and cost, and sidesteps the error rates that still plague image recognition.

The geographic robustness of the assay rests on both principle and evidence. Introduced populations typically harbor only a subset of source variation owing to founder effects, and continent-wide invasions of leaf-mining moths have repeatedly shown minimal or no mtDNA differentiation across the invaded range. Given insect mitochondrial substitution rates of ~1–2% per million years, de novo mutations arising and becoming fixed at a 20 bp primer site within a few years of an introduction are negligible. The newly available Kashmir records empirically support this expectation at the primer sites: all high-confidence Indian records share the European T state at the SXW-R 3′ terminus and show no substitution at the SXW-F site. Public sequence data also reveal BIN-level structure among Indian records: amplicon identity to the Italian PX980002 record reaches 99.626% in the BOLD:AAK9162 group but ranges from 95.888% to 98.318% in the BOLD:AFT3101 group. The evidence therefore supports primer-site conservation, not complete amplicon-wide identity among all Indian and European haplotypes. The single high-confidence primer-site dimorphism—the terminal 3′ base of the SXW-R site (C in the mitogenome references and validated Chinese populations; T in European and Indian field records)—has minimal functional impact because 3′-terminal C:A mismatches are among the best tolerated in PCR. Consistent with this expectation, the primers operate over a permissive annealing window (50–58 °C) with a detection limit ~1000-fold below the DNA content of a single adult, and both the additional Chinese populations and the synthetic T-haplotype template amplified cleanly.

### 4.2. Operational Advantages of the SS-COI Assay for Routine Laboratory Diagnostics

SS-*COI* primers target *COI* regions conserved in the target species but variable enough to discriminate congenerics [43]. A conventional PCR plus agarose gel—band present or absent—replaces sequencing, compressing the workflow to 2–3 h [44]. The approach has already been deployed for rapid identification of major pests in Lepidoptera [22,23,24,25,26], Coleoptera [31], Diptera [45], and Thysanoptera [46]. In this methodological lineage, “rapid molecular identification” has a well-established meaning: assays that bypass Sanger sequencing and de-liver a result within hours—as opposed to morphological identification, which requires experienced taxonomists and intact adult specimens, and standard DNA barcoding, which requires a sequencing platform and 1–2 working days. The present assay completes the extraction-to-result workflow in 2–3 h using only a thermal cycler and a gel rig, which is precisely what “rapid” denotes for SS-*COI* diagnostics.

Set against other platforms, the SS-*COI* system strikes a pragmatic balance of specificity, convenience, and cost for resource-limited settings. Standard barcoding depends on comprehensive reference databases, bidirectional sequencing, and bioinformatic expertise—requirements that inflate both turnaround time and expense [47]. LAMP removes the thermal cycler, yet multiple primer pairs in one reaction elevate non-specific pairing risk, and opening tubes for visual detection invites aerosol contamination and false positives [48,49]. RPA paired with lateral-flow strips enables visual readout, but core enzymes are costly, supply chains falter in remote areas, and false-positive rates are comparable [50]. The SS-*COI* assay here needs only a standard thermal cycler and gel rig—equipment already present at most grassroots plant-protection stations and port labs. No qPCR machine, sequencer, or disposable lateral-flow strips are required. We estimate the per-sample reagent cost of the complete assay at ≈14 CNY (≈US$2) in China, roughly one quarter of a LAMP reaction and without the sequencing fee and 1–2-day turnaround of standard barcoding.

The primers also tolerate a broad annealing range (50–58 °C). That thermal latitude insulates the reaction against cycler calibration drift, so different instruments in different locations still return reliable results—a real advantage for field deployment where infrastructure is patchy. Results are binary: target band present or absent. No fluorescence curves to interpret, no chromatograms to read. Any technician with basic PCR training can score the gel. Sealed reaction tubes plus negative and positive controls keep contamination risk low [51]. For large-scale screening, routine port monitoring, and emergency field diagnosis, SS-*COI* PCR is adaptable and scalable.

The operational setting further justifies the design. *L. malifoliella* is on the EPPO A1 List (since 2019) and on the A1 lists of Brazil (2018) and Chile (2019), and it is regulated as a quarantine pest in Canada (2019) and the United States (since 1995); it is also a target pest of the USDA Cooperative Agricultural Pest Survey program. With its ongoing range expansion into North Africa and South Asia, port inspectors and orchard-protection stations increasingly face specimens that morphology cannot resolve; these facilities are already equipped with thermal cyclers and gel electrophoresis, so their constraint is speed, specificity, and per-sample cost rather than instrument portability. The operational question at a port of entry is binary—does this interception contain the regulated pest *L. malifoliella*? A single-plex assay with positive and negative controls answers exactly that question with minimal cross-reactivity risk and unambiguous interpretation, mirroring official diagnostic protocols, which are structured as one protocol per regulated pest; virtually all published SS-*COI* assays are likewise single-plex. Multiplexing several pests into one reaction increases the risk of primer–primer interference and raises optimization costs, and given the low per-reaction price, parallel single-plex assays for two or three priority targets remain cheaper than one multiplexed or LAMP run. The assay therefore fills a genuine operational gap—a cheap, accurate, binary-readout laboratory diagnostic for quarantine screening—a role reflected in our companion study on the pest’s population dynamics and management in Chinese apple orchards.

### 4.3. Performance, Limitations, and Future Prospects of the Identification System

The assay performs robustly, rooted in the phylogenetic resolution of *COI* and the stringency of primer design [52]. K2P distances of 0.1508–0.1847 between *L. malifoliella* and the tested non-target species give the primers a clean discrimination margin. All field-collected samples validated at 100% accuracy. The 30 pg detection floor betters prior reports for *Tuta absoluta* (312.5 pg/μL) and *Phenacoccus manihoti* (135.2 pg/μL). That sensitivity is enough to amplify from a single egg or neonate larva, and to extract usable DNA from damaged port intercepts or early incipient populations. Primers amplify reliably across every life stage and tissue type tested—legs, antennae, head-thorax, abdomen, wings—so voucher specimens can remain intact while still yielding a firm identification. The same flexibility opens extensions such as predator gut-content analysis.

Still, the validation panel has gaps. Not every closely related *Leucoptera* or morphologically similar *Lyonetia* species was tested, so cross-reactions with unsampled taxa cannot be ruled out. Indian populations recently invaded, and Mediterranean and Central Asian populations carrying haplotypes not represented here, were not fully covered. Highly variable intraspecific sites falling within primer binding regions could, in principle, lower sensitivity or yield false negatives. Heavily degraded samples with template below 30 pg, or samples containing PCR inhibitors, also risk false negatives; adding an internal reference gene for quality control would address this [53]. It should nevertheless be stressed that no DNA-based method—SS-*COI*, LAMP, or qPCR alike—can guarantee a result from templates below its detection limit or from severely inhibited extracts; this is an inherent boundary of the analyte rather than a defect of this assay. The validated detection boundary of the present assay is 30 pg/μL, approximately one-thousandth of the DNA content of a single adult; severely degraded material yielding less template than this falls outside the validated range, and such samples should be re-extracted, concentrated, or routed to standard barcode sequencing as a fail-safe.

Other limitations are structural. The assay is single-target: one tube, one pest. It cannot simultaneously flag the suite of quarantine and economically important species that share apple orchards—codling moth (*Cydia pomonella*), oriental fruit moth (*Grapholita molesta*), *Phyllonorycter ringoniella*—in a single reaction. It still requires DNA extraction and thermal cycling, so it is not yet a true point-of-care test for the field or the inspection lane. Taxonomic and geographic coverage of the non-target panel needs expansion.

Future work can close these gaps. (1) Fold the *L. malifoliella* primers into a multiplex PCR to detect the major leaf-mining and fruit-boring apple pests in one tube, streamlining port screening of mixed intercepts. (2) Convert the primer sequences to LAMP or RPA paired with CRISPR-Cas12a detection, removing the thermal-cycler dependency and producing a visual field result [54,55]. (3) Systematically test populations across the Palaearctic, Oriental, and newly invaded regions to validate primer universality and flag any cryptic specificity issues. (4) Develop a qPCR version that quantifies target DNA alongside species confirmation, yielding semi-quantitative data for population-density monitoring and risk stratification [56]. (5) Link the molecular ID module to automated pheromone-trap networks to generate species-validated occurrence maps in real time, feeding directly into early-warning and precision-spray decision systems. These upgrades should move the assay from a laboratory workhorse to a genuinely field-deployable tool—one that gains value as *L. malifoliella* continues its geographic spread.

## 5. Conclusions

Morphology alone cannot reliably identify *Leucoptera malifoliella*: adults are too small, larvae are concealed, and related species overlap in character states. We developed a conventional PCR assay based on SS-*COI* primers (SXW-F/SXW-R) that amplifies a ~500 bp species-specific band from the target species and does not cross-react with five common leaf-mining apple pests. The primers amplify robustly across 50–58 °C annealing temperatures and work on all life stages from first-instar larva to adult, using DNA from antennae, head-thorax, abdomen, wings, or legs—ideal for damaged or partial specimens. Detection floor is ~0.03 ng/μL, better than most published SS-*COI* systems, and adequate for low-concentration field samples. The assay gives orchard managers, port inspectors, and IPM practitioners a fast, specific, and hardware-light molecular identification tool for *L. malifoliella*.

## Figures and Tables

**Table 1:** Collection information for the six Lepidoptera species used in this study.

No.	Species	*n*	Coordinates	Sampling Location	Date
1	*Adoxophyes orana*	30	113.89786, 38.43853	Shijiazhuang, Hebei	2024.9
2	*Cydia pomonella*	20	117.70747, 41.32971	Chengde, Hebei	2025.8
3	*Endothenia oblongana*	30	113.89786, 38.43853	Shijiazhuang, Hebei	2024.9
4	*Grapholita molesta*	30	113.89786, 38.43853	Shijiazhuang, Hebei	2024.9
5	*Leucoptera malifoliella*	50	113.89786, 38.43853	Shijiazhuang, Hebei	2025.8
6	*Phyllonorycter ringoniella*	30	113.89786, 38.43853	Shijiazhuang, Hebei	2024.9

## Data Availability

The *COI* barcode sequences generated in this study have been submitted to the NCBI GenBank database under the following accession numbers: PX456995 (*Adoxophyes orana*), PX456996 (*Cydia pomonella*), PX456997 (*Endothenia oblongana*), PX456998 (*Grapholita molesta*), PX456999 (*Leucoptera malifoliella*), and PX457000 (*Phyllonorycter ringoniella*). The raw experimental data supporting the findings of this study are available from the corresponding author upon reasonable request.

## Supplementary Materials

The following supporting information can be downloaded at: https://www.mdpi.com/article/10.3390/insects17080778/s1. Figure S1: Five other common microlepidopteran species in apple orchards. (A) *Phyllonorycter ringoniella*, (B) *Grapholita molesta*, (C) *Endothenia oblongana*, (D) *Adoxophyes orana*, (E) *Cydia pomonella.* Figure S2. Weekly monitoring results of five common microlepidopteran pests in apple orchards during July. (A) *Phyllonorycter ringoniella*, (B) *Grapholita molesta*, (C) *Adoxophyes orana*, (D) *Cydia pomonella*, (E) *Endothenia oblongana.* Figure S3. Adult body tissues of *Leucoptera malifoliella* used for evaluating the robustness of the SS-*COI* assay. (A) Antennae, (B) Wings, (C) Head and thorax (combined), (D) Legs, (E) Abdomen. Figure S4. (A) Neighbor-joining phylogenetic tree based on Kimura 2-parameter (K2P) distances of *COI* sequences from six lepidopteran species. Bootstrap values (1000 replicates) are indicated at the nodes. *Leucoptera malifoliella* forms a distinct monophyletic clade (bootstrap 93%) clearly separated from all non-target species. (B) Heatmap of pairwise K2P genetic distances among the six species. All interspecific distances exceed the 2% species delimitation threshold.
