# Supplementary material for: Species-Specific COI Primers for Rapid Molecular Identification of Leucoptera malifoliella
Source: Insects. 2026 Jul 28;17(8):778. doi: 10.3390/insects17080778 (PMC13513098; doi:10.3390/insects17080778)
Supplement: Supplementary file 1 [file insects-17-00778-s001.zip › insects-4415074-supplementary.pdf]

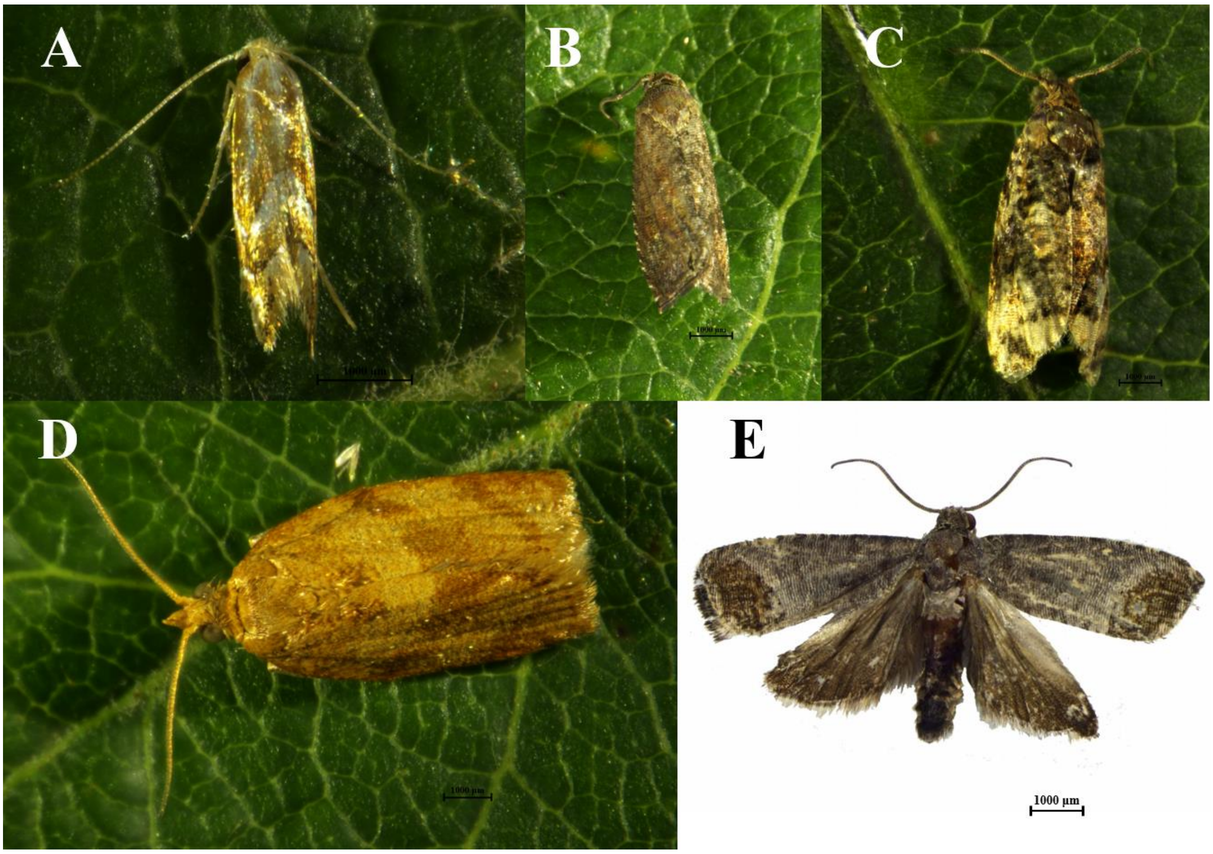

Figure S1. Five other common microlepidopteran species in apple orchards. (A) *Phyllonorycter ringoniella*, (B) *Grapholita molesta*, (C) *Endothenia oblongana*, (D) *Adoxophyes orana*, (E) *Cydia pomonella*.

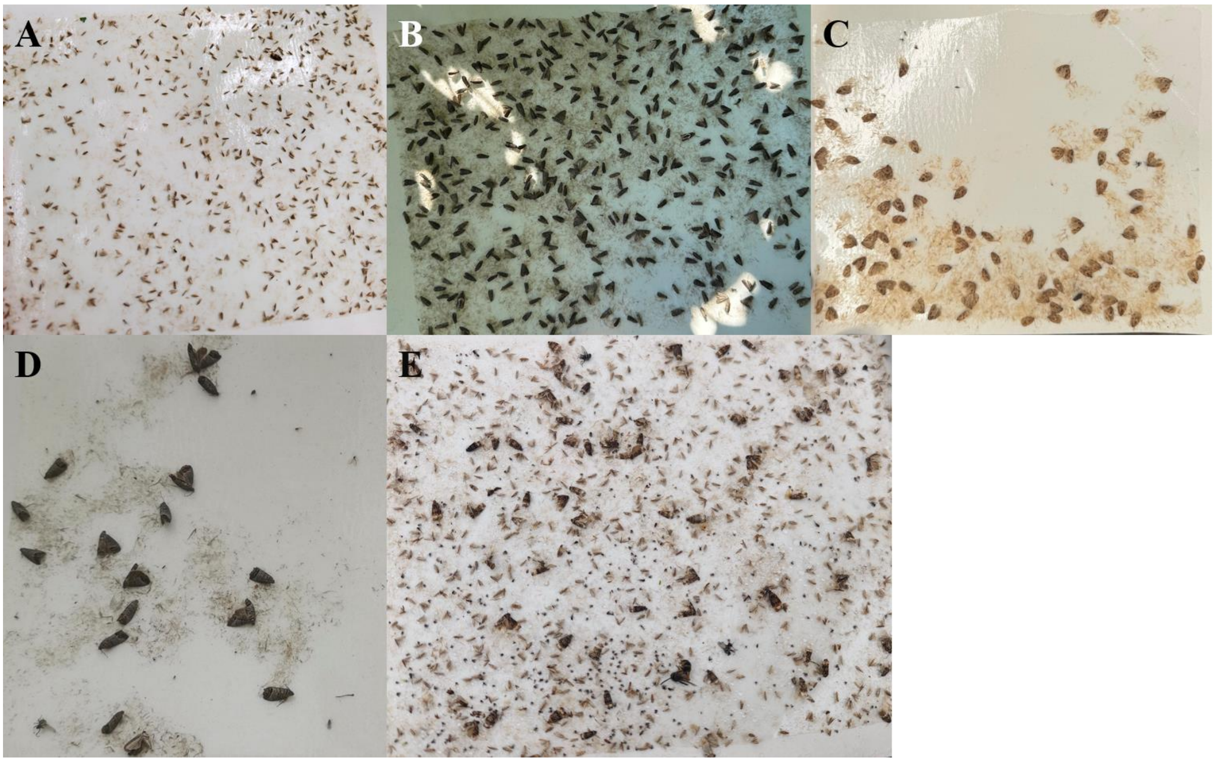

Figure S2. Weekly monitoring results of five common microlepidopteran pests in apple orchards during July. (A) *Phyllonorycter ringoniella*, (B) *Grapholita molesta*, (C) *Adoxophyes orana*, (D) *Cydia pomonella*, (E*) Endothenia oblongana*.

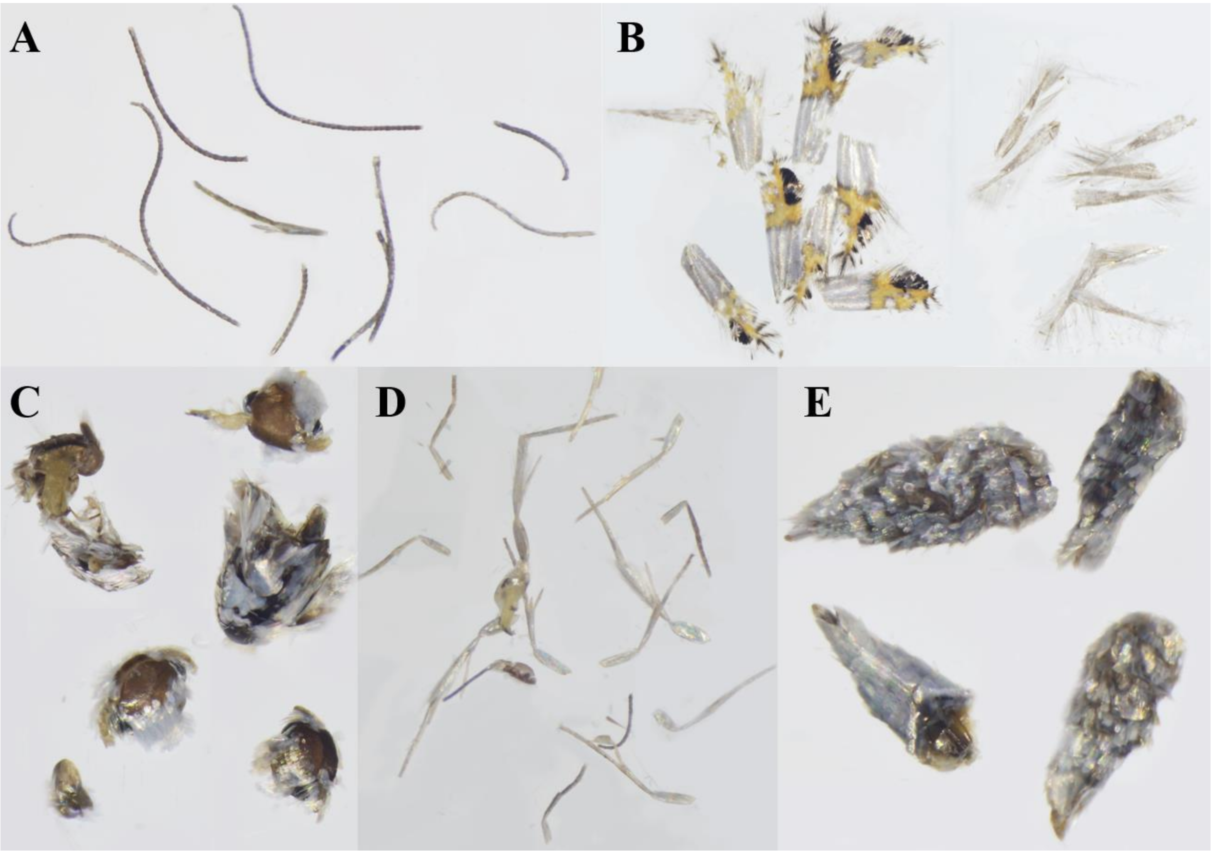

Figure S3. Adult body tissues of *Leucoptera malifoliella* used for evaluating the robustness of the SS-COI assay. (A) Antennae, (B) Wings, (C) Head and thorax (combined), (D) Legs, (E) Abdomen.

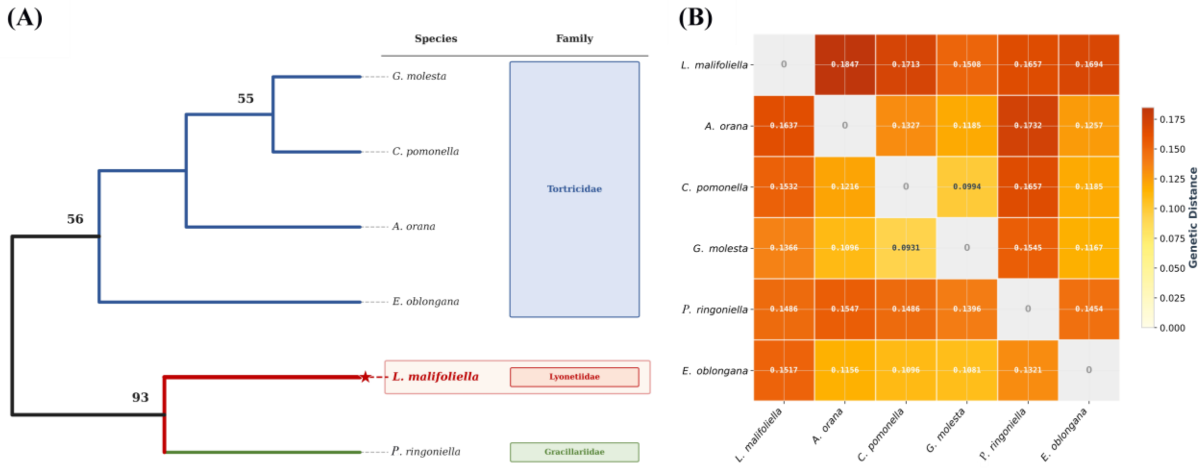

Figure S4. (A) Neighbor-joining phylogenetic tree based on Kimura 2-parameter (K2P) distances of COI sequences from six lepidopteran species. Bootstrap values (1,000 replicates) are indicated at the nodes. Leucoptera malifoliella forms a distinct monophyletic clade (bootstrap 93%) clearly separated from all non-target species. (B) Heatmap of pairwise K2P genetic distances among the six species. All interspecific distances exceed the 2% species delimitation threshold.
